# Methods for inferring neural circuit interactions and neuromodulation from local field potential and electroencephalogram measures

**DOI:** 10.1186/s40708-021-00148-y

**Published:** 2021-12-15

**Authors:** Pablo Martínez-Cañada, Shahryar Noei, Stefano Panzeri

**Affiliations:** 1 Neural Computation Laboratory, Istituto Italiano Di Tecnologia, Genova and Rovereto, Italy; 2 Optical Approaches To Brain Function Laboratory, Istituto Italiano Di Tecnologia, Genova, Italy; 3 CIMeC, University of Trento, Rovereto, Italy; 4 Department of Excellence for Neural Information Processing, Center for Molecular Neurobiology (ZMNH), University Medical Center Hamburg-Eppendorf (UKE), Hamburg, Germany

**Keywords:** Local field potential (LFP), Electroencephalogram (EEG), Neural oscillation, Information theory, Neural network model, Leaky integrate-and-fire (LIF) neuron model, Neuromodulation

## Abstract

Electrical recordings of neural mass activity, such as local field potentials (LFPs) and electroencephalograms (EEGs), have been instrumental in studying brain function. However, these aggregate signals lack cellular resolution and thus are not easy to be interpreted directly in terms of parameters of neural microcircuits. Developing tools for a reliable estimation of key neural parameters from these signals, such as the interaction between excitation and inhibition or the level of neuromodulation, is important for both neuroscientific and clinical applications. Over the years, we have developed tools based on neural network modeling and computational analysis of empirical data to estimate neural parameters from aggregate neural signals. This review article gives an overview of the main computational tools that we have developed and employed to invert LFPs and EEGs in terms of circuit-level neural phenomena, and outlines future challenges and directions for future research.

## Introduction

Neural activity is often recorded at the level of aggregate electrical signals. These signals are recorded invasively in animals (for example, local field potentials, LFPs, and electrocorticograms, ECoGs [1, 2]) or non-invasively in humans (for example, electroencephalograms, EEG, and magnetoencephalograms, MEG [1, 3–5]). These different aggregate brain signals largely share the same neural sources and have major applications in both scientific research and clinical diagnosis. They are easy to record, capture many circuit-level aggregate phenomena, including key synaptic integrative signals at different organization levels from mesoscopic to macroscopic brain scales, and can reveal oscillatory activity over a wide range of frequencies [1, 2, 6–9]. However, neural aggregate signals are more difficult to interpret than spiking activity of individual neurons, because they conflate and add together contributions from many complex neural processes [1, 2, 6–8]. It is therefore notoriously difficult to link them to individual neural circuit features. For example, we still cannot interpret simple modulations of EEG/LFP features, such as a change in LFP or EEG oscillatory power, in terms of excitation, inhibition, and their interaction. This hinders us from understanding cognitive computations in humans and animals, understanding the neural underpinnings of brain disorders, and developing effective interventions. Being able to separate contributions of different neural phenomena to LFPs or EEGs, and to quantify how neural parameters change with manipulations of neural circuits or in brain disorders, will enhance our understanding of how best to use LFPs or EEGs to study brain function and dysfunction.

Over the years, we have developed numerous computational tools to address this challenge. Our approach includes advanced methods to identify meaningful bands in the frequency domain in neural recordings, neural network models to predict key neural phenomena, and computationally guided perturbations of neural activity to causally validate model predictions. This paper summarizes progress achieved by our lab in the interpretation of aggregate electrical signals and introduces new directions and challenges for future research in this field. Since this is an extended review of our work presented as Plenary Talk at the 14th International Conference on Brain Informatics BI 2021 [10], here we have principally focused on describing the computational methods and the results coming from our own Laboratory. We would like, though, to remind the readers of the large number of very important contributions in this field made by many other authors, summarized in recent important reviews [1, 2, 5, 11, 12].

## Cortical oscillations and their role in neural computation

Much of our work has been aimed at understanding the neural mechanisms and functions for information processing of brain oscillations captured by LFPs and EEGs. We thus briefly describe some basic features of neural oscillatory activity that are relevant for our review.

Aggregate electrical signals recorded in the cerebral cortex often display prominent oscillatory activity. A large bulk of evidence shows that oscillations seen in neural activity are not simply an epiphenomenon, but are a core mechanism in a variety of cognitive, sensory and information transmission functions [4, 13–24]. Synchronization of neuronal oscillations at different frequencies is a pervasive feature of neuronal activity and is thought to facilitate the transmission and integration of information in the cerebral cortex. Neural aggregate signals have been thus decomposed and interpreted in the frequency domain [1, 6, 8]. Traditionally, neural oscillations have been divided into canonical frequency bands such as the widely used delta (1–4 Hz), theta (4–8 Hz), alpha (8–12 Hz), beta (15–30 Hz) and gamma (30–100 Hz) bands. Associations robustly found between band-limited power signals and distinct behavioral states or sensory inputs strongly support the validity of this approach [6, 23, 25–27].

Gamma-band oscillations have received much attention in the last few decades [13–15, 20, 21, 24, 28]. There is a general acceptance that gamma oscillations reflect the interaction between excitation and inhibition in local cortical circuits [20, 21, 29–31]. The power of gamma oscillation encodes information about sensory stimuli, motor and cognitive variables [4, 23, 24, 32–40]. It has been shown that gamma oscillations are also implicated in facilitating or modulating inter-areal or within-area communication [4, 18, 21, 41–46]. Moreover, and of particular importance for the interpretation of neuroimaging experiments in humans, the gamma band is the frequency band that correlates the most with the functional magnetic resonance imaging (fMRI) signal [47, 48]. The slower theta, alpha and beta rhythms have been involved in many cognitive functions. These slower oscillations have been proposed to mediate top-down perceptual decision processes, encoded in long-range cortical inputs, which could also interact with gamma-band synchronization [4, 20, 24, 49]. Thus, several cortical rhythms coexist in the cerebral cortex, which are often nested into each other and cooperate to shape brain functions and neuronal information processing [20].

## Analytical methods to identify regions of the frequency spectrum capturing different neural phenomena of interest

Numerous studies have characterized the role of the different frequency bands in brain function. However, the individuation and definitions of the exact boundaries of individual frequency bands are often largely arbitrary, based on heuristic criteria and vary substantially between studies [2]. Thus, a first major problem when trying to infer neural mechanisms from aggregate signals is to provide an objective approach to separate aggregate neural signals into different bands each reflecting a different neural phenomenon, and to establish a correspondence between specific frequency regions of the LFP or EEG power spectrum and the underlying neural mechanisms.

**Fig. 1:**
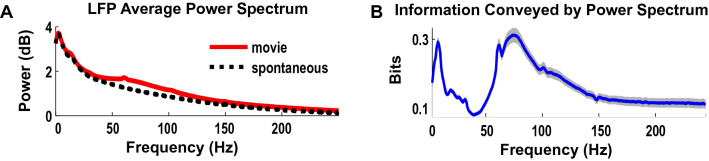
Comparison of power and information spectra. Data were taken from primary visual cortex of anaesthetized macaques during stimulation with naturalistic movies. **A** Power spectrum. **B** Information conveyed by power spectrum. Recomputed from data first published in [23, 48]

One difficulty in this endeavor is that the average neural power spectrum (over either time epochs or trials) of a typical recording (see Fig. 1A for an example of LFP recordings in visual cortex during naturalistic stimulation) is dominated by a power-law aperiodic component and often lacks easily identifiable oscillatory peaks [23, 50]. This could lead us to think that there is no distinctive structure in the power spectrum and, thus, there is no possibility for a clear and objective separation in frequency bands. However, the average spectrum may mask individual variations that correspond to different processing modalities or functions, especially for complex tasks or during stimulation with naturalistic sensory stimuli.

**Fig. 2:**
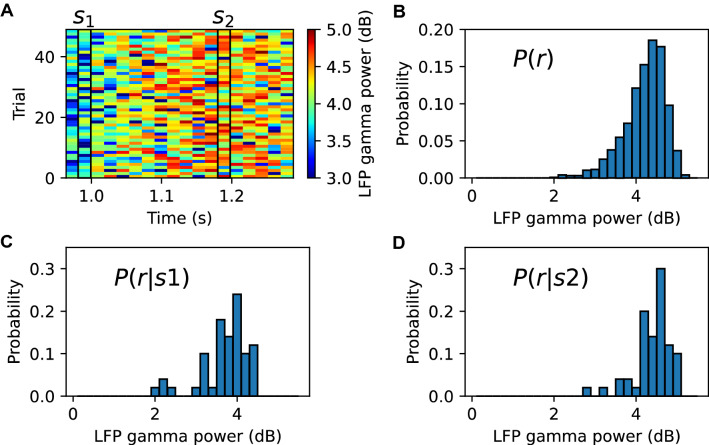
Illustration of computation of the mutual information carried by LFP power about movie scenes. **A** Simulation of single-trial LFP power in the gamma band (from 70 to 80 Hz) using a sparsely connected recurrent network of excitatory and inhibitory neurons [40]. To simulate periods of low and high LFP power, which approximate the different movie scenes used in the original publication [40], we modulated the external input rate of the model by superposition of a sine wave with frequency 1 Hz and a constant rate signal. The spectrogram was computed over half a cycle of the sinusoid. Every time window of the spectrogram was considered a different scene $s$ ($s_{1}$ and $s_{2}$ are a period of low and high LFP gamma power, respectively). Thus, the probability of each scene $P\left( s \right)$ is the inverse of the number of time windows. **B** Probability distribution $P\left( r \right)$ of the LFP gamma power across all trials and scenes. Probability distribution $P\left( {r|s} \right)$ of the LFP gamma power across all trials given the presented scenes $s_{1}$ (**C**) and $s_{2}$ (**D**)

To capture how individual Fourier frequencies vary their power over time in relation to stimulus variations, we developed an information theoretic algorithm (illustrated in Fig. 2) that quantifies the amount of information about each possible stimulus that is carried by the LFP power at a given frequency [40]. The theoretical foundations of information theory (see [51, 52]) demonstrate that mutual information is the best measure to capture all possible ways in which a neural signal can carry information about any sensory variable of interest. To create the stimulus set, we divided the presentation time of the movie into different time windows (Fig. 2A), each considered a different stimulus $s$ (in other words, a different movie scene). We computed the information between the stimulus window in the movie $s$ that was being presented and the power of the LFP at a given frequency $f$, as follows:

$$I\left( {S;R_{f} } \right) = \mathop \sum \limits_{s} P\left( s \right)\mathop \sum \limits_{{r_{f} }} P\left( {r_{f} |s} \right)\log_{2} \frac{{P\left( {r_{f} |s} \right)}}{{P\left( {r_{f} } \right)}},$$

where $P\left( s \right)$ is the probability of presentation of the stimulus window $s$ (here, this is the inverse of the total number of time windows in which we divided the movie sequence), $P\left( {r_{f} |s} \right)$ is the probability of observing a power $r_{f}$ at a frequency $f$ in response to the stimulus $s$ in a single trial (Fig. 2C and D), and $P\left( {r_{f} } \right)$ is the probability of observing the power $r_{f}$ across all trials in response to any stimulus (Fig. 2B).

To facilitate the sampling of response probabilities, the space of power values at each frequency was binned [23]. Information is non-negative and quantifies the average reduction of the uncertainty about the stimulus that can be gained from observing a single-trial neural response. We measured it in units of bits, one bit corresponding to a reduction of uncertainty by a factor of two. Importantly, the quantification of information based on the division of the movie into stimulus windows or scenes without defining which visual feature (e.g., contrast, orientation, etc.) is represented in each movie frame allowed us to capture all information about any possible visual features (including both static image features and their variation from frame to frame) present in the movie.

The information spectrum computed for V1 data during naturalistic stimulation showed a clear structure that was invisible in the average spectrum: there were only two bands that carried stimulus information, a low-frequency (1–8 Hz) and a high-frequency gamma band (60–100 Hz), whereas middle frequencies carried little information (Fig. 1B). It is important to note that the gamma band has been traditionally implicated in the coding of information about specific visual features, such as orientation or contrast of a visual input [35, 37]. The low-frequency band, to our knowledge, was not implicated in the coding of visual information in V1 by any previous study. This discovery of an extra information channel not considered before was in our view enabled by two key features. First, from an experimental point of view, it was crucial to use a complex dynamics visual stimulus (a movie) that included not only a rich variety of image features from one frame to the next, but also a rich variety of naturalistic temporal dynamics of those features. Second, from a computational point of view, we used a formalism that accounted for all possible sources of information, and, in this way, allowed us to identify the sources of coding of information and neural pathways not considered before. This gives us an example of the potential of using an information theoretic analysis for discovering channels that carry different kinds of neural information and thus need to be included in models as partly different neural pathways.

Extending the information theory approach to the multivariate case of information carried by pairs of frequencies (see [53]) allowed us to characterize specific regions of the information spectrum as belonging to only one or multiple bands. This partition into functionally meaningful bands can be achieved very precisely (and even to the point of individuating the optimal frequency values determining the boundaries between different bands) by quantifying patterns of redundancy or independence between the information carried by different frequencies [48]. For example, if the information carried by one frequency is independent of amplitude variations in another frequency, then these two frequencies probably capture different neural contributions to the LFP. If the two frequencies carry redundant information instead, they likely originate from common neural phenomena. Application of this approach to visual cortical data has revealed three different functional bands in the information spectrum [23]. Frequencies in the gamma (60–100 Hz) range exhibited high visual information and had large redundancy among them, indicating that neural responses at these frequencies have a common component that is stimulus-driven. The same applies to low frequencies (1–8 Hz), where there was high redundancy between frequencies. Importantly, low and high-frequency frequencies carried independent information, indicating that they act as independent visual information channels and probably originate from separate neural processes.

Finally, frequencies between 15 and 38 Hz exhibited high correlations between them but not with stimulus information. Based only on these results of the information theoretic analysis, we hypothesized that signals in this middle frequency range are generated by a common process unrelated to the visual stimuli—for example, a neuromodulatory input [23]. We will discuss in the Section “Perturbation experiments guided by predictions of computational models to study the effect of neuromodulation on cortical oscillations” how this hypothesis could be tested causally by pharmacological intervention.

Importantly, the principles of information theory can be used to understand not only how information is encoded in the oscillatory power or phase of each frequency band, but also how activity in different bands is involved in transmission of information across different neural populations. We used the same recordings of LFP activity in macaque V1 during natural movie stimulation discussed above. Our information theoretic methods (in particular, directed measures of information transfer such as transfer entropy) allowed us to investigate how oscillations of cortical activity in the gamma frequency band may influence dynamically the direction and strength of information flow across different groups of neurons. We found that the local phase of gamma-band rhythmic activity exerted a stimulus-modulated and spatially asymmetric directed effect on the firing rate of spatially separated populations within the primary visual cortex [45]. The relationships between gamma phases at different sites could be described as a stimulus-modulated gamma-band wave propagating along the spatial directions with the maximal flow of information transmitted between neural populations. We observed that gamma waves changed direction during presentation of different movie scenes, and when this occurred, the strength of information flow in the direction of the gamma wave propagation was transiently reinforced. Given that travelling gamma waves indicated the direction of causation in neural activity, we hypothesized that these shifts were associated to a propagation of gamma oscillations along the horizontal connections of V1. Interestingly, we found support for this hypothesis from the fact that the properties of gamma waves were compatible with known physiological and anatomical properties of lateral connectivity. First, travelling gamma waves had an average propagation speed (approximately 364 cm/s) that was similar in magnitude to the signal propagation speed along axons of excitatory horizontal connections reported in the literature [54–57]. Second, information transfer mediated by gamma waves was quantitatively stronger among pairs with similar orientation preference, compatible with the finding that horizontal connections are more likely among populations with similar orientation preferences [58–60]. These effects were specific to the gamma band and were not found in other low-frequency bands [45]. These results suggest that traveling gamma waves mark and causally mediate the dynamic reconfiguration of functional connections and the transfer of visual information within V1 [45].

Together, these examples show the power of information theoretic approaches to interpret individual frequencies in terms of variations with stimuli or behavioral state and to identify a minimal set of meaningful bands whose origin can then be investigated with the aid of computational models and perturbation experiments, as we illustrate in the next sections.

## Mathematical modeling of neural network dynamics

### Neural network models to identify neural mechanisms for information encoding

**Fig. 3:**
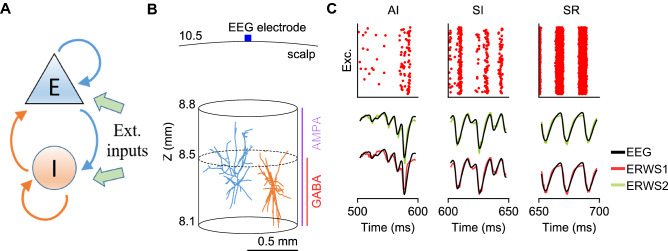
**A** Recurrent inhibitory–excitatory (I–E) network of LIF point neurons. Excitatory and inhibitory neurons receive two different types of external inputs: a sensory-driven input and a cortico-cortical input. **B** Network of multicompartment neuron models used in the hybrid modeling approach [72, 73] to compute the ground-truth EEG signal. **C** Raster plots of spiking activity (top panels) of the LIF network model for the asynchronous irregular (AI), synchronous irregular (SI) and synchronous regular (SR) network states. Comparison between ground-truth EEGs and outputs of the current-based ERWS1 and ERWS2 proxies (bottom panels)

The above information theoretic analysis individuated two frequency bands that were shown to carry different channels of visual information. The question that arises is what neural circuit mechanisms are expressed by each band. To address this question, we developed a formalism based on fitting recurrent network models of interacting excitatory and inhibitory point neurons (Fig. 3A) to data. These models reduce the morphology of neurons to a single point in space and their dynamics are described by a set of coupled differential equations that can be solved efficiently numerically and often also analytically. Despite their simplicity, these models have been widely used to describe important properties of cortical microcircuits [61], such as sensory information coding [40, 62], working memory [63, 64], attention [65] or sleep slow waves [66]. In particular, we developed a recurrent network model of leaky integrate-and-fire (LIF) neuronal populations composed of 5000 neurons. Consistent with the ratio of excitatory and inhibitory neurons found in the cerebral cortex, 4000 neurons were excitatory (i.e., their projections onto other neurons formed AMPA-like excitatory synapses) and 1000 inhibitory (i.e., their projections formed GABA-like synapses), randomly connected with a connection probability between each pair of neurons of 0.2. All neurons in the model receive external inputs (both a sensory-driven thalamic input and a noisy intracortical input) to predict some key aspects of neural activity in primary visual cortex during naturalistic visual stimulation and spontaneous activity [40, 62, 67].

Specifically, in ref. [40, 62], we found that by studying this simulated network we could capture the translation rules between stimulus dynamics and LFP frequency bands. Confirming theoretical results that showed that gamma power in a recurrent network tends to increase with the strength of the input to the network [30], we found that the network encoded the overall strength of the input into the power of gamma-band oscillations generated by inhibitory–excitatory neural interactions. In addition, we found that the network encoded slow dynamic features of the input into slow LFP fluctuations mediated (through entrainment to the inputs) by stimulus–neural interactions. Thus, our recurrent network model could provide evidence for the dual encoding of information in both the low-frequency information channel (carrying temporal information of the dynamics of sensory-driven thalamic inputs) and the gamma-band information channel (reflecting excitatory inhibitory interactions modulated by the strength of thalamic inputs). Interestingly, the model also reproduced other higher order features of the dynamics of visual cortex, including the independence of the information carried by low- and high-frequency information channels when using naturalistic visual stimuli [23], and the cross-frequency coupling between the EEG delta-band phase and gamma-band amplitude [67].

However, our model [40, 62, 67] could not reproduce the excess in power and the strong within-band correlations observed in real data for the mid-range (19–38 Hz) band in visual cortex [23]. Our model did not include changes in neural activity induced by neuromodulation, further corroborating the idea that stimulus-independent neuromodulatory factors are needed to model the dynamics of this mid-range band.

### Realistic computation of field potentials from point-neuron network models

The above studies compared qualitatively and quantitatively information patterns in neural network models and real data to make inferences about which neural pathway contribute to each frequency band. As demonstrated in ref. [30], this question can be addressed even without having to compute a realistic LFP or EEG from the network models, because basic oscillation properties of the network can be observed both at the level of spiking activity of neurons and at the level of aggregate signals.

We have then begun to investigate the more difficult problem of trying to measure, or to infer, the precise value of microscopic neural parameters, such as the activity of individual classes of neurons within a network, from aggregate activity measures such as EEG or LFP. To obtain a more precise estimation of network parameters, it is necessary to compute a realistic LFP or EEG from these network models of point neurons. However, these neuron models lack a spatial structure, which prevents modelers from being able to compute the spatially separated transmembrane currents that are necessary to generate LFPs and EEGs in real biological networks.

In our initial studies [40, 62, 67], we estimated the LFP and EEG based on the sum of absolute values of synaptic currents from simulation of the network model. Other studies have proposed different approaches to compute extracellular potentials using other variables of the simulation, such as the average membrane potentials [66, 68], the average firing rate [30, 69] or the sum of all synaptic currents [70, 71].

We then evaluated systematically [72, 73] the limitations and caveats of using such ad hoc simplifications to estimate the LFP or EEG from neuron models without spatial structure (i.e., point-neuron models). We compared how well different approximations of field potentials (termed proxies) proposed in the literature reconstructed a ground-truth signal obtained by means of the hybrid modeling approach [72, 74] (Fig. 3B). This approach includes a network of unconnected multicompartment neuron models with realistic three-dimensional (3D) spatial morphologies. Each multicompartment neuron is randomly assigned to a unique neuron in the network of point neurons and receives the same input spikes of the equivalent point neuron. Since the multicompartment neurons are not connected to each other, they are not involved in the network dynamics and their only role is to transform the spiking activity of the point-neuron network into a realistic estimate of the LFP or EEG that is used as the ground-truth signal against which we compared different candidate proxies (Fig. 3C).

We found that a specific weighted sum of synaptic currents from the point-neuron network model, for a specific network state (i.e., asynchronous irregular), performed remarkably well in predicting the LFP [72]. We then extended our study to the EEG [73] by including a head model that approximated the different geometries and electrical conductivities of the head necessary for computing a realistic EEG signal recorded by scalp electrodes. We chose the four-layered spherical head model [75, 76] that included different layers that represented the brain tissue, cerebrospinal fluid (CSF), skull, and scalp.

We also validated our EEG proxies across the repertoire of network states displayed by recurrent network models [30, 77], namely the asynchronous irregular (AI), synchronous irregular (SI), and synchronous regular (SR) (Fig. 3C). The states generated by the LIF neuron network were produced by systematically varying across simulations the firing rate of the thalamic input ($\upsilon_{0}$) and the relative strength between inhibitory and excitatory synapses ($g = g_{{\text{I}}} /g_{{\text{E}}}$). The validation of our proxies for a wide range of values of $g$ and $\upsilon_{0}$ is important to solve the inverse modeling approach and to ensure that our proxies can be used to robustly predict these network parameters from the varied shapes of experimentally recorded EEGs (see Sect. 4.3).

We found that a new class of linear EEG proxies, based on a weighted sum of synaptic currents, outperformed previous approaches and worked well under a wide range of network configurations with different cell morphologies, distributions of presynaptic inputs and positions of the EEG electrode. We also evaluated whether our proxies could perform well when combined with a more complex and anatomically detailed human head model: the New York head model [78], which takes into account the folded cortical surface of the human brain. The EEG topographic maps calculated by applying our proxies to the New York head model correctly predicted time traces of the EEG signal at different electrode positions.

### Changes in excitation–inhibition (E/I) balance in simulated neural aggregate signals

**Fig. 4:**
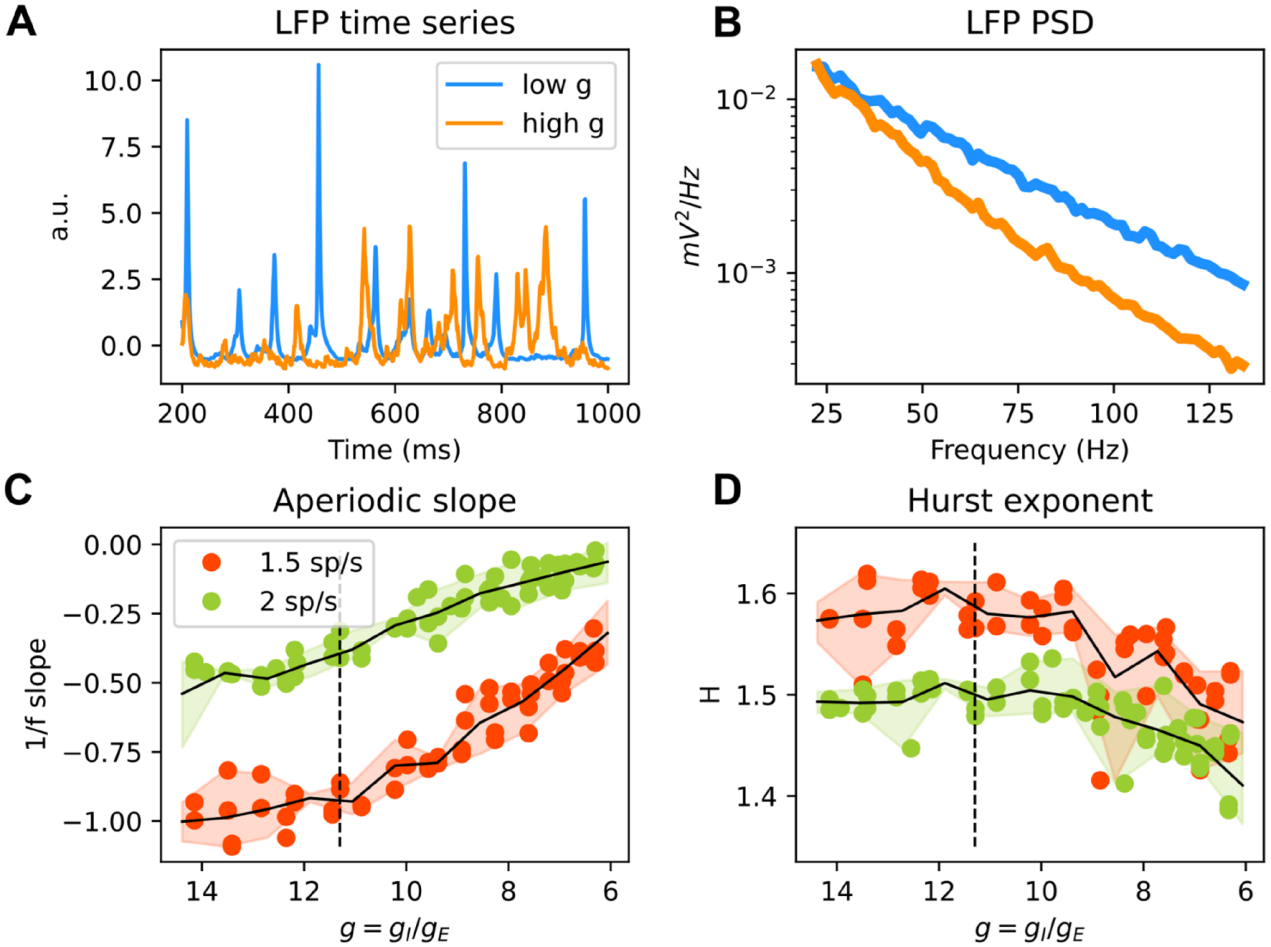
LFPs (**A**) and PSDs (**B**) generated for two different ratios between inhibitory and excitatory conductances ($g = g_{I} /g_{E}$). The relationship between 1/f slopes (**C**) and Hurst exponents (**D**) are plotted as a function of $g$ for two different firing rates of thalamic input (1.5 and 2 spikes/second). The reference value of $g$ (which has shown in previous studies to reproduce cortical data well) is represented by a dashed black line. Recomputed and replotted from data published in ref. [79]

Our realistic estimations of aggregate signals from simple point-neuron networks allowed us to invert and use these models to estimate some neural parameters of circuit activity that are not directly accessible from the EEG and LFP. For example, we considered how we could use network models to estimate from such recordings the ratio between excitation and inhibition. The theory of neural network models [30] and the empirical electrophysiological data have reported that the E/I ratio has profound effects on the spectral shape of neural activity. Its imbalance has been implicated in neuropsychiatric conditions, including Autism Spectrum Disorder. In ref. [79], we investigated different biomarkers computed on the power spectrum of LFPs and fMRI blood oxygen level-dependent (BOLD) signal that could be used to reliably estimate the E/I ratio. These biomarkers were the exponent of the 1/f spectral power law, slopes for the low- and high-frequency regions of the spectrum and the Hurst exponent (H). We simulated the LFP (Fig. 4A) and BOLD signal from our recurrent network model, and studied how these biomarkers changed when we manipulated the E/I ratio by independently varying the strengths of the inhibitory ($g_{{\text{I}}}$) and excitatory ($g_{{\text{E}}}$) synaptic conductances [80]. Part of our results are shown in Fig. 4. A flattening of 1/f slopes (Fig. 4C) was found in the excitation-dominated region where the E/I ratio is shifted in favor of E than the reference value used previously [40, 62, 67] to capture cortical power spectra. We also observed that H decreased in the excitation-dominated region (Fig. 4D). However, shifting the E/I balance towards stronger inhibition had a weaker effect on slopes and H. We then validated our model against in vivo chemogenetic manipulations in mice that either increased neurophysiological excitation or silenced the local activity in the network. When modeling effects of chemogenetic manipulations within the recurrent network model, we found that DREADD manipulations that enhanced excitability of pyramidal neurons reduced steepness of the slopes and led to a decrease in H. Then, we used the predictions of our model of how the ratio $g$ between inhibition and excitation affects spectral properties such as slopes and H (see Fig. 4) to interpret the spectra of resting state fMRI (rsfMRI) in the medial prefrontal cortex (MPFC) of subjects within the autism spectrum disorder. We found that H was reduced in the MPFC of autistic males but not females, and using our model we interpreted this change in spectral properties as an indicator of increased excitation in males.

## Perturbation experiments guided by predictions of computational models to study the effect of neuromodulation on cortical oscillations

Biophysically realistic computational models and information theoretic methods can be used to generate predictions and test them with suitably designed perturbation experiments. Finding the best strategy to do it is an active topic of research. In what follows, we briefly review our attempts to address this challenge.

As reviewed above, based on our information theoretic analysis, we have proposed that the mid-frequency range (15–38 Hz approx.), which exhibited high correlations within frequency bands but contained little visual information, may reflect a single source of neuromodulatory inputs. We designed a perturbation experiment to test this hypothesis [81]. We recorded the LFP in primary visual cortex (V1) of anesthetized macaques during spontaneous activity and during visual stimulation with naturalistic movies while pharmacologically perturbing dopaminergic neuromodulation by systemic injection of L-DOPA (a metabolic precursor of dopamine). We found that dopaminergic neuromodulation had marked effects on both spontaneous and movie-evoked neural activity. During spontaneous activity, dopaminergic neuromodulation increased the power of the LFP specifically in the 19–38 Hz band, suggesting that the power of endogenous visual cortex oscillations in this band can be used as a robust marker of dopaminergic neuromodulation. These results confirmed the hypothesis that we made in earlier work [23] based on information theoretic analysis of field potentials. Moreover, dopamine increased visual information encoding over all frequencies during movie stimulation. The information increase due to dopamine was prominent in the supragranular layers of visual cortex that project to higher cortical area and in the gamma band of LFP power spectrum that has been previously implicated in mediating feedforward information transfer. We concluded that dopamine may promote the readout of relevant sensory information by strengthening the transmission of information from primary to higher areas [81].

These observations, which in our view could not have been made by either computational analyses or blind design of perturbation experiments alone, illustrate the power of effectively combing them.

## Conclusion

Understanding the microcircuit dynamics and computations underlying EEG and LFP features has the potential to allow researchers to make fundamental discoveries about brain function and to effectively use measures extracted from aggregate electrical signals as a reliable biomarker of brain pathologies. In this paper, we have reviewed our approach based on computational modeling and advanced analytical tools of neural network dynamics to interpret neural aggregate signals in terms of neural circuit parameters. We have developed tools to partition the LFP and EEG power spectrum into different meaningful frequency bands and to identify frequency channels and neural pathways that process largely independent and different kinds of neural information. We have shown preliminary work on estimating neural circuit parameters, such as excitation, inhibition and their interaction, from aggregate neural signals. Here we outline some limitations of our approach and the major challenges that we must address in the future.

In Refs. [72, 73] we have developed accurate LFP and EEG proxies that open up the possibility of computing realistic EEG and LFP predictions from simple network models. These predictions can be then compared to empirical EEGs and LFPs and can be used to estimate neural model parameters. However, to achieve this goal we still need to accomplish several steps. First, we need to develop statistical tools that can infer neural parameters (such as the ratio between excitation and inhibition or properties of network connectivity) from EEG and LFP spectral features by fitting such models to empirically measured spectra. Then, we need to carefully validate the statistical inference approaches on real brain data in which neural circuit parameters can been manipulated by the experimenter, for example by means of chemogenetic manipulations [79]. We could validate the inference algorithm by studying if it is able to predict the type of controlled manipulation that has been applied in each dataset (e.g., whether a manipulation produced an increase or decrease of the E/I balance).

Although we used realistic modeling of neurons and networks, our models do not capture the full complexity of the brain. It would be particularly important to extend our models to include different classes of neurons, such as different types of interneurons. We could include inter-areal interactions between different recurrent networks to generate wider oscillation ranges than the gamma oscillations mostly considered in our work, which would be useful to study the relationship between local oscillations and functional connectivity [82]. It would also be important to model the effects of different kinds of neuromodulators on distributed processing.

In previous work [81], we developed methods to study the effect of global and diffuse patterns of neuromodulation. However, an emergent view [83] is that neuromodulation can be non-global and depend on target specificity and the differentiated spatiotemporal dynamics within brain stem nuclei. It will be important to implement analytical tools to identify first individual ensembles in the locus coeruleus (LC) and to understand then how neural activity of these LC ensembles drive cortical states [84].

Given the above limitations, and although more work is needed to be able to interpret empirical aggregate signals such as EEGs and LFPs in terms of network model parameters and neuromodulation, we expect that future research can build on the encouraging results presented in this paper and lead to a credible, robust and biologically plausible estimation of neural parameters from neural aggregate signals.

## Data Availability

This is a review that contains no new data. Software for simulation of neural network models of spiking point neurons and multicompartment neurons and for computation of EEG proxies can be found at https://github.com/pablomc88/EEG_proxy_from_network_point_neurons. Software for the information theoretic calculations can be found at https://sicode.eu/results/software.html.

## Abbreviations

AI: Asynchronous irregular
BOLD: Blood oxygen level-dependent
ECoG: Electrocorticogram
EEG: Electroencephalogram
fMRI: Functional magnetic resonance imaging
LC: Locus coeruleus
LIF: Leaky integrate-and-fire
LFP: Local field potential
MEG: Magnetoencephalogram
MPFC: Medial prefrontal cortex
rsfMRI: Resting state fMRI
SI: Synchronous irregular
SR: Synchronous regular
